# Supplementary material for: Comparative genomics provides new insights into the diversity, physiology, and sexuality of the only industrially exploited tremellomycete: Phaffia rhodozyma
Source: BMC Genomics. 2016 Nov 9;17:901. doi: 10.1186/s12864-016-3244-7 (PMC5103461; doi:10.1186/s12864-016-3244-7)
Supplement: Additional file 6: — List of orphan genes with links to PFAM (related to Additional file 1: Table S1). (ZIP 1428 kb) [file 12864_2016_3244_MOESM6_ESM.zip › BLAST_HTML_FTR/G00513_P.html]

BLAST Search Results


```
BLASTP 2.2.27+


Reference:
Stephen F. Altschul, Thomas L. Madden, Alejandro A. Schäffer,
Jinghui Zhang, Zheng Zhang, Webb Miller, and David J. Lipman (1997),
"Gapped BLAST and PSI-BLAST: a new generation of protein database
search programs", Nucleic Acids Res. 25:3389-3402.


Reference for
composition-based statistics:
Alejandro A. Schäffer, L. Aravind, Thomas L. Madden, Sergei
Shavirin, John L. Spouge, Yuri I. Wolf, Eugene V. Koonin, and
Stephen F. Altschul (2001), "Improving the accuracy of PSI-BLAST
protein database searches with composition-based statistics and
other refinements", Nucleic Acids Res. 29:2994-3005.


Database: nr
           71,551,133 sequences; 26,053,659,533 total letters


Query= G00513_P

Length=606
                                                                      Score     E
Sequences producing significant alignments:                          (Bits)  Value

emb|CED83823.1|  HMG-box transcription factor [Xanthophyllomyces ...  1194    0.0  
ref|XP_006457446.1|  hypothetical protein AGABI2DRAFT_196139 [Aga...  50.8    0.003
gb|KJA17647.1|  hypothetical protein HYPSUDRAFT_70519 [Hypholoma ...  50.8    0.004
gb|KDR73234.1|  hypothetical protein GALMADRAFT_251818 [Galerina ...  47.8    0.033
gb|KIL65264.1|  hypothetical protein M378DRAFT_178427 [Amanita mu...  45.8    0.11 
gb|KIK00820.1|  hypothetical protein K443DRAFT_678919 [Laccaria a...  45.8    0.13 
gb|KDQ51363.1|  hypothetical protein JAAARDRAFT_50784 [Jaapia arg...  44.7    0.29 
gb|EMD41279.1|  hypothetical protein CERSUDRAFT_109878 [Ceriporio...  43.9    0.47 


 >emb|CED83823.1| HMG-box transcription factor [Xanthophyllomyces dendrorhous]
Length=727

 Score = 1194 bits (3090),  Expect = 0.0, Method: Compositional matrix adjust.
 Identities = 597/598 (99%), Positives = 597/598 (99%), Gaps = 0/598 (0%)

Query  8    VTRDFYSSHALALKATFQSRYPAYEYTRRPNNTRKRGSRGGKTYISRRDSSSWKSPDDTS  67
            VTRDFYSSHALALKATFQSRYPAYEYTRRPNNTRKRGSRGGKTYISRRDSSSWKSPDDTS
Sbjct  130  VTRDFYSSHALALKATFQSRYPAYEYTRRPNNTRKRGSRGGKTYISRRDSSSWKSPDDTS  189

Query  68   ASTVLGGSALGLERMNPRNQDGSSGWNRNGYSIGETEQDSDTDTNKHDNDGSSSSQYSDH  127
            ASTVLGGSALGLERMNPRNQDGSSGWNRNGYSIGETEQDSDTDTNKHDNDGSSSSQYSDH
Sbjct  190  ASTVLGGSALGLERMNPRNQDGSSGWNRNGYSIGETEQDSDTDTNKHDNDGSSSSQYSDH  249

Query  128  SQGNRPASSIHSELPTPQLPYYPLTTEYGPPNELSSSAPAYYKPEITSFSSMSAATSASA  187
            SQGNRPASSIHSELPTPQLPYYPLTTEYGPPNELSSSAPAYYKPEITSFSSMSAATSASA
Sbjct  250  SQGNRPASSIHSELPTPQLPYYPLTTEYGPPNELSSSAPAYYKPEITSFSSMSAATSASA  309

Query  188  TTHNNSSGTYGHSPHVFVSPYGWRANRSRPVSTSLMGSSPYSPSSSLSSFSPQLSVNGRL  247
            TTHNNSSGTYGHSPHVFVSPYGWRANRSRPVSTSLMGSSPYSPSSSLSSFSPQLSVNGRL
Sbjct  310  TTHNNSSGTYGHSPHVFVSPYGWRANRSRPVSTSLMGSSPYSPSSSLSSFSPQLSVNGRL  369

Query  248  HGHGKVPVENGTNQTYTAATQDYPVPRASSRRPDALIEPSPASQSQQLATATSTADPSAP  307
            HGHGKVPVENGTNQTYTAATQDYPVPRASSRRPDALIEPSPASQSQQLATATSTADPSAP
Sbjct  370  HGHGKVPVENGTNQTYTAATQDYPVPRASSRRPDALIEPSPASQSQQLATATSTADPSAP  429

Query  308  SFHSSALYQPSARDWSTDPFRRHSVQRARTGPLHALGFLKSEQQQLQQSSSYKWTESDSH  367
            SFHSSALYQPSARDWSTDPFRRHSVQRARTGPLHALGFLKSEQQQ QQSSSYKWTESDSH
Sbjct  430  SFHSSALYQPSARDWSTDPFRRHSVQRARTGPLHALGFLKSEQQQPQQSSSYKWTESDSH  489

Query  368  HRPHSDPAVEDESRSEGGYRAGGSQAESRGWNSTYQEESTYPPAHAQSHLHSHSQSYTHS  427
            HRPHSDPAVEDESRSEGGYRAGGSQAESRGWNSTYQEESTYPPAHAQSHLHSHSQSYTHS
Sbjct  490  HRPHSDPAVEDESRSEGGYRAGGSQAESRGWNSTYQEESTYPPAHAQSHLHSHSQSYTHS  549

Query  428  HFPSDQMHAYSPLPPLATTATSIEPSSTMSSAPIGVPSTSGLLKYSNNVYPSHSHSQSLI  487
            HFPSDQMHAYSPLPPLATTATSIEPSSTMSSAPIGVPSTSGLLKYSNNVYPSHSHSQSLI
Sbjct  550  HFPSDQMHAYSPLPPLATTATSIEPSSTMSSAPIGVPSTSGLLKYSNNVYPSHSHSQSLI  609

Query  488  STSAPTTSLSPFTYSSYQQNTHHLQPLARTSALSAPKTEYPGSFVDQSSYHSSGSDTVHR  547
            STSAPTTSLSPFTYSSYQQNTHHLQPLARTSALSAPKTEYPGSFVDQSSYHSSGSDTVHR
Sbjct  610  STSAPTTSLSPFTYSSYQQNTHHLQPLARTSALSAPKTEYPGSFVDQSSYHSSGSDTVHR  669

Query  548  VGPFDQDHMADRVRDSQRHSYQTDPQTHAKYESWSAGVGVEANARDRTPMMDSYYYES  605
            VGPFDQDHMADRVRDSQRHSYQTDPQTHAKYESWSAGVGVEANARDRTPMMDSYYYES
Sbjct  670  VGPFDQDHMADRVRDSQRHSYQTDPQTHAKYESWSAGVGVEANARDRTPMMDSYYYES  727


>ref|XP_006457446.1| hypothetical protein AGABI2DRAFT_196139 [Agaricus bisporus var. 
bisporus H97]
 ref|XP_007331799.1| hypothetical protein AGABI1DRAFT_108046 [Agaricus bisporus var. 
burnettii JB137-S8]
 gb|EKM77527.1| hypothetical protein AGABI1DRAFT_108046 [Agaricus bisporus var. 
burnettii JB137-S8]
 gb|EKV41835.1| hypothetical protein AGABI2DRAFT_196139 [Agaricus bisporus var. 
bisporus H97]
Length=445

 Score = 50.8 bits (120),  Expect = 0.003, Method: Compositional matrix adjust.
 Identities = 48/161 (30%), Positives = 65/161 (40%), Gaps = 19/161 (12%)

Query  10   RDFYSSHALALKATFQSRYPAYEYTRRPNNTRKRGSRGGKTYISRRDSSSWKSPDDTSAS  69
            + FY   A  LK  F S+YP Y Y RRPNNTRK+  RG    + + D+      DD    
Sbjct  120  KQFYLDQAKILKENFNSKYPDYVYRRRPNNTRKKNRRGDIGGLRQLDNPQL---DDIGGH  176

Query  70   TVLGGSALGLERMNPRNQDGSSGWNRNGYSIGETEQDSDTDTNKHDNDGSSSSQYSDHSQ  129
             +   S    E ++P +                   D     + H   G + S+YS  S 
Sbjct  177  EIGESSHTDDEVLDPHSH------------YPRVPHDLPHAYDNHARYGPAPSRYSIPSD  224

Query  130  GNRPASS----IHSELPTPQLPYYPLTTEYGPPNELSSSAP  166
            G RP S+    I  + P+ +LP        G PN  S S P
Sbjct  225  GFRPPSTHDTRIQYQQPSDRLPQDIAGARLGQPNHSSYSYP  265


>gb|KJA17647.1| hypothetical protein HYPSUDRAFT_70519 [Hypholoma sublateritium 
FD-334 SS-4]
Length=468

 Score = 50.8 bits (120),  Expect = 0.004, Method: Compositional matrix adjust.
 Identities = 26/55 (47%), Positives = 31/55 (56%), Gaps = 9/55 (16%)

Query  10   RDFYSSHALALKATFQSRYPAYEYTRRPNNTRKRGSRGGKTYISRRDSSSWKSPD  64
            + FY  HA  LK TF SRYP Y Y RRPNN+RKR          R D+   +SP+
Sbjct  123  KQFYLEHAKQLKETFNSRYPDYVYRRRPNNSRKR---------RRSDAGQLRSPE  168


>gb|KDR73234.1| hypothetical protein GALMADRAFT_251818 [Galerina marginata CBS 
339.88]
Length=473

 Score = 47.8 bits (112),  Expect = 0.033, Method: Compositional matrix adjust.
 Identities = 24/43 (56%), Positives = 27/43 (63%), Gaps = 4/43 (9%)

Query  1    MPSQKTTVTRDFYSSHALALKATFQSRYPAYEYTRRPNNTRKR  43
            MPS +    + FY   A  LK TF SRYP Y Y RRPNN+RKR
Sbjct  115  MPSSE----KQFYLEQAKQLKETFNSRYPDYVYRRRPNNSRKR  153


>gb|KIL65264.1| hypothetical protein M378DRAFT_178427 [Amanita muscaria Koide 
BX008]
Length=457

 Score = 45.8 bits (107),  Expect = 0.11, Method: Compositional matrix adjust.
 Identities = 20/37 (54%), Positives = 24/37 (65%), Gaps = 0/37 (0%)

Query  10   RDFYSSHALALKATFQSRYPAYEYTRRPNNTRKRGSR  46
            + FY   A  LK TF S+YP Y Y RRPNN+RK+  R
Sbjct  118  KQFYLDRAKQLKETFNSKYPDYVYRRRPNNSRKKSKR  154


>gb|KIK00820.1| hypothetical protein K443DRAFT_678919 [Laccaria amethystina LaAM-08-1]
Length=489

 Score = 45.8 bits (107),  Expect = 0.13, Method: Compositional matrix adjust.
 Identities = 20/34 (59%), Positives = 23/34 (68%), Gaps = 0/34 (0%)

Query  10   RDFYSSHALALKATFQSRYPAYEYTRRPNNTRKR  43
            + FY   A  LK TF +RYP Y Y RRPNN+RKR
Sbjct  124  KQFYLDQAKHLKETFNTRYPDYVYRRRPNNSRKR  157


>gb|KDQ51363.1| hypothetical protein JAAARDRAFT_50784 [Jaapia argillacea MUCL 
33604]
Length=472

 Score = 44.7 bits (104),  Expect = 0.29, Method: Compositional matrix adjust.
 Identities = 25/57 (44%), Positives = 34/57 (60%), Gaps = 3/57 (5%)

Query  10   RDFYSSHALALKATFQSRYPAYEYTRRPNNTR-KRGSRGGKTYISRRDSSSWKSPDD  65
            + FY   A  LK  F S+YP Y Y RRPNN+R KR + GG + ++  DS+S   P +
Sbjct  112  KQFYLDQAKKLKDNFNSKYPDYVYRRRPNNSRKKRRTEGGPSRLA--DSASTLDPGE  166


>gb|EMD41279.1| hypothetical protein CERSUDRAFT_109878 [Ceriporiopsis subvermispora 
B]
Length=529

 Score = 43.9 bits (102),  Expect = 0.47, Method: Compositional matrix adjust.
 Identities = 26/75 (35%), Positives = 37/75 (49%), Gaps = 12/75 (16%)

Query  10   RDFYSSHALALKATFQSRYPAYEYTRRPNNTRKRGSRGGKTYISRRDSSSWKSPDDTSAS  69
            + FY   A  LK  F S+YP Y Y RRPNN+RK+          R+  +  +SP D S  
Sbjct  120  KKFYLDQAKKLKDNFNSKYPDYVYRRRPNNSRKK----------RKPDAGQQSPTDASPP  169

Query  70   TVLGGSALGLERMNP  84
              +    + LE ++P
Sbjct  170  GEIDD--MSLEELSP  182


Lambda      K        H        a         alpha
   0.307    0.121    0.356    0.792     4.96 

Gapped
Lambda      K        H        a         alpha    sigma
   0.267   0.0410    0.140     1.90     42.6     43.6 

Effective search space used: 6654239111748


  Database: nr
    Posted date:  Sep 23, 2015 12:05 AM
  Number of letters in database: 26,053,659,533
  Number of sequences in database:  71,551,133


Matrix: BLOSUM62
Gap Penalties: Existence: 11, Extension: 1
Neighboring words threshold: 11
Window for multiple hits: 40
```
